# Immune Response of Multiparous Hyper-Immunized Sows against Peptides from Non-Structural and Structural Proteins of PRRSV

**DOI:** 10.3390/vaccines3040973

**Published:** 2015-11-27

**Authors:** Edgar Rascón-Castelo, Alexel Burgara-Estrella, Mónica Reséndiz-Sandoval, Andrés Hernández-Lugo, Jesús Hernández

**Affiliations:** 1 Laboratorio de Inmunología, Centro de Investigación en Alimentación y Desarrollo A.C (CIAD) Carretera a la Victoria Km 0.6, Hermosillo, Sonora 83304, Mexico; E-Mails: erascon83@gmail.com (E.R.-C.); alexel.burgara@gmail.com (A.B.-E.); mresendiz@ciad.mx (M.R.-S.); 2 Consultoría Agropecuaria del Noroeste, Hermosillo, Sonora 83106, Mexico; E-Mail: ahernandez_lugo@hotmail.com

**Keywords:** PRRSV, non-structural proteins, structural proteins, peptides, IFN-γ secreting cells

## Abstract

The purpose of this study was to evaluate the humoral and cellular responses of commercial multiparous and hyper-immunized sows against peptides from non-structural (nsp) and structural proteins of porcine reproductive and respiratory syndrome virus (PRRSV). We selected sows with different numbers of parities from a commercial farm. Management practices on this farm include the use of the MLV commercial vaccine four times per year, plus two vaccinations during the acclimation period. The humoral response was evaluated via the antibody recognition of peptides from nsp and structural proteins, and the cellular response was assessed by measuring the frequency of peptide and PRRSV-specific IFN-gamma-secreting cells (IFNγ-SC). Our results show that sows with six parities have more antibodies against peptides from structural proteins than against peptides from nsp. The analysis of the cellular response revealed that the number of immunizations did not affect the frequency of IFNγ-SC and that the response was stronger against peptides from structural proteins (M protein) than against nsp (nsp2). In summary, these results demonstrate that multiparous, hyper-immunized sows have a stronger immune humoral response to PRRSV structural peptides than nsp, but no differences in IFNγ-SC against the same peptides were observed.

## 1. Introduction

Porcine reproductive and respiratory syndrome (PRRS) is a disease characterized by reproductive failure in sows, respiratory distress, growth reduction and high mortality [1,2,3]. PRRS is currently considered the most significant and economically devastating disease impacting the swine industry worldwide. This disease is responsible for losses of approximately $664 million US per year in the USA [4]. Therefore, the pork industry and scientific community are striving to control and prevent this disease.

The etiologic agent of PRRS is the porcine reproductive and respiratory syndrome virus (PRRSV). PRRSV was initially identified in Europe [2] and later in the USA [1]. PRRSV is a member of the *Arterivirus* genus, *Arteriviridae* family and *Nidovirales* order, along with the equine arteritis virus, lactate dehydrogenase elevating virus and simian hemorrhagic fever virus [5]. The PRRSV genome is approximately 15 kb in length and contains at least 10 open reading frames (ORFs). ORF1a and 1b comprise approximately 80% of the viral genome and encode two polyproteins that, after enzymatic cleavage, result in 14 non-structural proteins (nsp) involved in virus replication and the regulation of the immune response [3]. Additionally, the virus expresses eight structural proteins, GP2, E, GP3, GP4, GP5, M, N, and 5a that are encoded by the ORF2a, ORF2b, ORF3 through ORF7 and ORF5a genes, respectively [3,5].

The majority of the PRRSV-infected pigs develop an immunity that is eventually able to control and eliminate the infection and can protect against homologous re-infections, but this immunity is not able to completely protect against a heterologous challenged. However, the precise mechanism responsible for inducing the protection remains unknown. Neutralizing antibodies and IFN-γ are the most studied immune mechanisms against PRRSV; however, these components are not solely responsible for PRRSV immunity [6]. Multiple antigens have also been tested as vaccine candidates, but there is currently no single antigen that induces cross-protective and long-term immunity. However, the use of commercial vaccines is a common practice to control PRRSV infection. Different reports have shown that vaccination reduces viremia and complications associated with PRRSV infection [7,8,9], but protection against heterologous field viruses are deficient [9,10]. Efforts to improve vaccine efficacy in the field include the use of a large number of vaccinations [11,12], but other reports have suggested that two vaccinations are sufficient to induce protective immunity [13,14]. The aim of this study was evaluate the antibodies and frequency of IFN-γ secreting cells (IFNγ-SC) specific for peptides from nsp and structural proteins of PRRSV present in multiparous and hyper-immunized sows, based on the hypothesis that the number of parities, each of which is associated with additional immunizations, increases the immune response. Our results showed that multiparous, hyper-immunized sows have a stronger response against structural peptides, but the frequency of IFNγ-SC against the same peptides was not different between sows with different number of parities and vaccine applications.

## 2. Experimental Section

### 2.1. Animals

Blood samples were collected from a commercial pig production farm located in the northwest region of Mexico. The majority of the samples was obtained in April 2013, and a smaller subset was obtained in October 2013. The production system was farrow-to-finish, the sow population was 2500, and the sows were primarily F1 Landrace × Yorkshire crossbreeds. Sows were housed in individual stalls in early gestation, group pens in late gestation and in farrowing crates during lactation. The sow vaccination program was as follows. Quarantine: PRRS MLV, swine influenza virus, *Actinobacillus pleuropneumoniae*, *Mycoplasma hyopneumoniae*, porcine circovirus 2 and ReproCyc^®^ PLE (twice each one). Prior to farrow: *Mycoplasma hyopneumoniae*, swine influenza virus and *E. coli*. Lactation: ReproCyc^®^ PLE. The farm was PRRSV-positive but without outbreaks in the last 6 years (last outbreak winter 2009). Whole-herd vaccination to sows with Ingelvac^®^ PRRS-MLV (Boeringher-Ingelheim Vetmedica Inc. St. Joseph, MO, USA) was performed every 3 months (since 2009 to 2013), and two applications were given to gilts during the acclimation phase. According to farm records, sows with one parity had received lower numbers of Ingelvac^®^ PRRS-MLV vaccinations in comparison to sows with six parities (Table 1 and Table 2).

**Table 1:** Characteristics of sows included in this study.

Identification	Number of Parities	Age in Years	No. of Immunizations ^a^
4829	1	1.15	3 to 4
4920	1	1.16	3 to 4
4880	1	1.16	3 to 4
4796	1	1.16	3 to 4
4674	1	1.16	3 to 4
4467	1	1.16	3 to 4
4498	1	1.21	3 to 4
3573	3	2.01	7 to 8
3291	3	2.04	7 to 8
3638	3	1.94	7 to 8
3703	3	1.92	7 to 8
3643	3	2.00	7 to 8
3635	3	1.96	7 to 8
4078	3	ND ^b^	7 to 8
3641	3	2.44	7 to 8
3722	3	2.39	7 to 8
3146	4	2.80	9 to 11
3643	4	2.48	9 to 11
2231	5	3.26	11 to 12
2388	5	3.21	11 to 12
1771	6	3.08	11 to 13
1639	6	3.25	11 to 13
2038	6	2.87	11 to 13
1704	6	3.13	11 to 13
1937	6	2.81	11 to 13
1778	6	3.08	11 to 13
1744	6	2.89	11 to 13

^a^ the number of immunizations was calculated according to information in Table 2; ^b^ ND, data was unavailable.

**Table 2:** Farm vaccination program and estimation of immunizations/sow/parity.

Year	Week ^a^	No. of Immunizations ^b^	Age ^c^	Note
2010	4	1	1	Begin massive vaccination program
	17	2	1.3	
	30	3	1.6	Twice vaccination of sows with six parities in quarantine^d^
	43	4	1.9	
2011	4	5	2	
	17	6	2.3	
	30	7	2.6	
	43	8	2.9	
2012	4	9	3	
	17	10	3.3	
	30	11	3.3	Twice vaccination of sows with one parity in quarantine
	43	12	3.9	
2013	4	13	4	
	17	14	4.3	Last vaccination of experimental sows
	30	15	4.6	
	43	16	4.9	
2014	4	17	5	End of massive vaccination program

^a^ production week in one year; ^b^ farm program of massive immunization with PRRS MLV vaccine; ^c^ approximation of sow age; ^d^ the time of the first two immunizations of sows during quarantine. According this approximation, sows with six parities evaluated in this study received at least 13 immunizations.

### 2.2. Peptides

Eighteen peptides previously described as IFN-γ inducers were used in this study (Table 3) [15,16,17,18]. Peptides were synthetized by GenScript (Piscataway, NJ, USA) at a crude purity and adjusted to a concentration of 1 mg/mL in water. All peptides were used at final concentration of 0.5 µg/mL for peptide-ELISA. For the IFN-γ ELISPOT assay, the peptides were used at a final concentration of 10 µg/mL in the single peptide solution and in the pooled peptides solution.

**Table 3:** Peptides used in this study.

Protein		Peptide Sequence	Amino Acid Position	Genotype	Reference
**Non-Structural Proteins**	nsp2-SV	SLYKLLLEV	589–597	I	[18]
	nsp2-WL	WLFAGVVLL	1141–1149	I	[18]
	nsp3-YL	YIWHFLLRL	1337–1345	I	[18]
	nsp5-LV	LLNEILPAV	1929–1937	I	[18]
	nsp5-IL	IIIGGLHTL	2025–2033	I	[18]
	nsp5-IV	ILNEVLPAV	2046–2054	I	[18]
**Structural Proteins**	Gp4-FI	FLLAGAQHI	7–15	I	[17]
	Gp4-CT	CLFAILLAT	175–183	I	[17]
	Gp5-CR	CAFAAFVCFVIR	117–128	I	[17]
	Gp5-LC	LAALICFVIRLAKNC	117–131	II	[16]
	Gp5-KK	KGRLYRWRSPVIVEK	149–163	II	[16]
	Gp5-TP	TPLTRVSAEQWGRP	187–200	II	[18]
	M-CS	CNDSTAPQKVLLAFS	9–23	II	[15]
	M-AL	ALKVSRGRLLGLLHL	33–47	II	[15]
	M-FV	FGYMTFAHFESTNRV	57–71	II	[15]
	M-KK	KFITSRCRLCLLGRK	93–107	II	[15]
	N-IE	IRHHLTQTE	64–72	II	[17]
	N-VT	VRLIRVTST	113–121	II	[17]

### 2.3. IDEXX ELISA

Antibodies against PRRSV were detected using a commercial ELISA kit (Herdchek Porcine Reproductive and Respiratory Syndrome Antibody Test Kit, IDEXX Laboratories, Westbrook, Maine) according to the manufacturer’s directions.

### 2.4. Peptide ELISA

Sera samples were used to detect peptide-specific antibodies as reported by de Lima *et al.* [15]. Briefly, 96-well EIA/RIA clear flat bottom polystyrene high-binding microplates (Corning, Inc., New York, NY, USA) were coated with a peptide solution (0.5 µL/mL per peptide) in 0.1 M carbonate buffer, pH 9.6. After coating, the microplates were blocked with 300 µL per well of PBS containing 0.01% Tween-20 (PBST-20) and 10% wt. non-fat dry milk solution for 4 h at room temperature on a plate shaker. After three washes with 300 µL of PBST-20 solution, 100 µL of serum diluted 1:20 with 5% wt. non-fat dry milk in PBST-20 was added and incubated at 37 °C for 1 h. After washing, 100 µL of goat anti-porcine IgG monoclonal Ab labeled with peroxidase (dilution 1:2000) (Southern Biotech Associates, Inc., Birmingham, AL, USA) was added and incubated for 1 h at room temperature. Finally, 50 µL of TMB (Sigma-Aldrich, St. Louis, MO, USA) was added and incubated for 15 min at room temperature in the dark, and the reaction was stopped with 50 µL of H_2_SO_4_ and read on a spectrophotometer at 450 nm. Sera from naïve pigs were used as negative controls.

### 2.5. PBMC Isolation

Peripheral blood mononuclear cells (PBMCs) from hyper-immunized sows were collected into heparin-coated tubes (Becton-Dickinson, BD, Franklin Lakes, NY, USA). PBMCs were separated from whole blood by density-gradient centrifugation with Ficoll-Hypaque (GE Healthcare Life Sciences, Uppsala, Sweden) following the manufacturer’s instructions. Thereafter, the PBMCs were washed three times in RPMI-1640 with antibiotics and resuspended in complete RPMI-1640 medium supplemented with 2 mM l-glutamine, 1 mM sodium pyruvate, 100 IU penicillin, 1 mg/mL amphotericin B solution (all from Sigma-Aldrich) and 10% fetal bovine serum (Gibco, Life Technologies, Grand Island, NY, USA).

### 2.6. IFN-γ ELISPOT Assay

IFN-γ ELISPOT assays were performed according to Díaz *et al.* [17] with some modifications. Briefly, high-binding 96 well EIA/RIA plates (3590, Corning Incorporated, Corning, NY, USA) were coated with a commercial capture antibody against porcine IFN-γ (Porcine IFN-γ P2G10, BD Biosciences Pharmingen, San Jose, CA, USA) and diluted in sterile 0.05 M carbonate-bicarbonate buffer at pH 9.6. The plates were incubated at 4 °C overnight prior to the isolation of PBMCs. After this incubation, each well was washed two times with sterile PBS and once with RPMI-1640 medium without fetal bovine serum (FBS). The plate was then incubated with complete RPMI-1640 for 2 h at 37 °C in a 5% CO_2_ atmosphere. PBMCs were dispensed and stimulated for up to 20 h with a single peptide or a mixture of peptides (10 µg/mL) or with homologous (MVL vaccine virus) or heterologous (strain NVSL 97-7895, GenBank accession No. AY545985) PRRSV at a m.o.i. of 0.1. Unstimulated PBMCs and PHA-stimulated (10 μg/mL) cells were included as negative and positive controls, respectively. After 20 h of incubation, each well was washed with PBS containing 0.01% Tween-20 (Sigma-Aldrich). The detection antibody, biotin mouse anti-pig IFN-γ (P2C11, BD Biosciences Pharmingen), was dispensed into the wells, and the ELISPOT assay was then revealed by the addition of TMB membrane peroxidase substrate (T0565, Sigma-Aldrich). The results were expressed as the frequency of peptide-specific IFNγ-SC per 1 × 10^6^ PBMCs.

### 2.7. Statistical Analysis

The significance of the variability among the groups was determined by one-way analysis of variance (ANOVA) followed by Tukey’s test using GraphPad PRISM software Version 5.02 (GraphPad, La Jolla, CA, USA). The level of statistically significant difference was set at *p* < 0.05.

## 3. Results

### 3.1. Sows

We evaluated sows from a commercial farm that used the Ingelvac^®^ PRRS-MLV vaccine to control PRRSV. All gilts were vaccinated twice during acclimation, and then all sows were vaccinated four times per year every year (Table 1 and Table 2). We chose sows with different numbers of parities based on the premise that sows with more parities had received more vaccinations; thus we hypothesized that a difference in the immunity level could be detected. To estimate the number of immunizations, farm records such as the day of birth, movement to quarantine, and parity were used. According to Table 2, sows with one parity have had between three and four immunizations; sows with three parities have had between seven and eight; and sows with six parities have had at least eleven immunizations (Table 2).

The number of piglets born alive, stillborn pigs, mummified fetuses and piglets at weaning were compared between parity and with farm records. In 2013, the overall mean number of piglets born alive at the farm was 11.2, and the mean numbers of piglets were 9.1, 11.4 and 12.4 in sows with one, three and six parities, respectively. The mean number of stillborn piglets at the farm was 0.35, and means of 0.2, 0.4 and 0.4 were found in sows with one, three and six parities, respectively. The overall mean number of mummified fetuses at the farm was 0.39, and means of 0.1, 0.1 and 0.2 were found in sows with one, three and six parities, respectively. Finally, the overall mean number of piglets at weaning was 10.19 at the farm, and means of 9.5, 9.8 and 10.2 were found in sows with one, three and six parities, respectively. In general, there were no significant differences between parity of when compared with records from the farm.

### 3.2. Antibody Response

**Figure 1:**
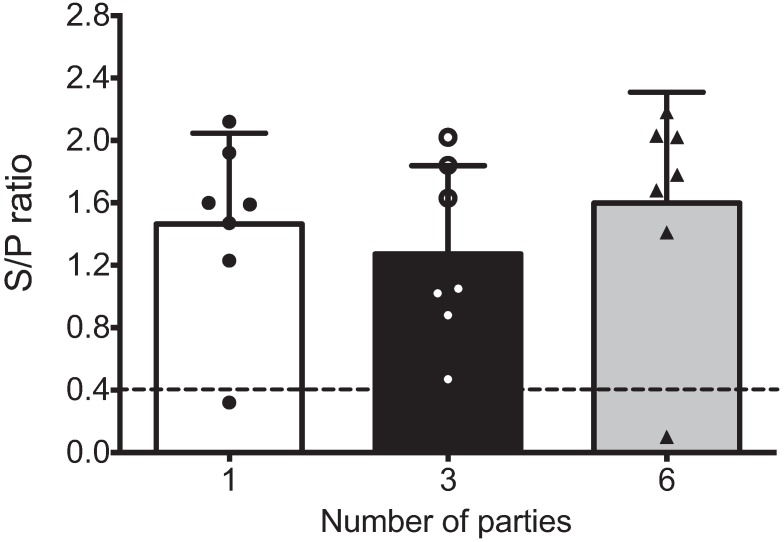
Seropositivity of multiparous and hyper-vaccinated sows against PRRSV. PRRSV-specific antibodies were detected in the sera of sows based on parity groups using IDEXX ELISAs (*n* = 7 per group). The positive threshold was a sample to positive (s/p) ratio of 0.4, according to the manufacturer’s instructions. No statistical significance was observed between the groups in this assay. Each bar represents the S/P ratio mean of seven sows ± standard deviation. White, black and gray bars represent the groups of sows with one, three and six parities, respectively.

Sows were grouped according to their number of parities (one, three or six), and the antibody response against PRRSV was evaluated to confirm seropositivity. Positive samples were those with S/P values ≥ 0.4. Figure 1 shows that the mean S/P values in all groups were >1.2 (S/P range from 0.470 to 2.180), and no significant differences were observed. Only two sows were seronegative, one with a single parity and one with three parities.

**Figure 2:**
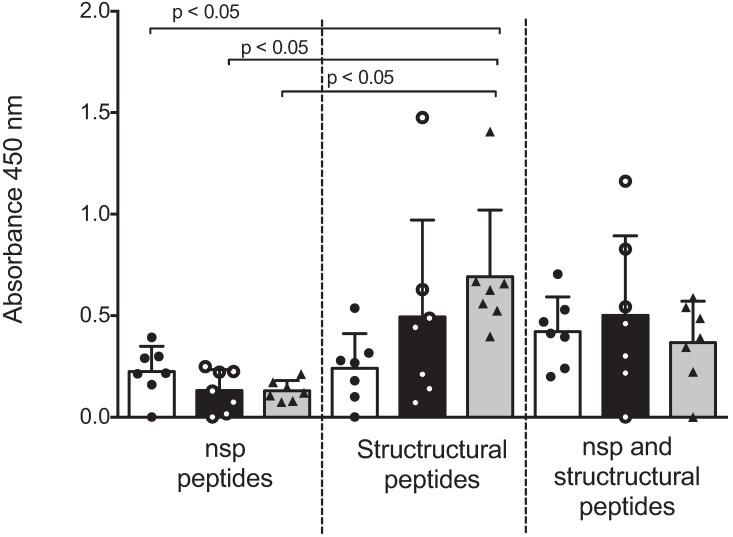
Antibody recognition of IFN-γ inducer peptides by multiparous and hyper-vaccinated sows. Humoral response in multiparous sows with the nsp peptides and structural protein peptides of PRRSV were detected in sera samples from sows (*n* = 7 per group) by indirect ELISA. These data represent one independent experiment from a total of three with similar results and are expressed as the mean OD. Each bar represents the mean of the OD values of the sow sera ± standard deviation. Independent OD values were adjusted against the OD value from negative pig sera. White, black and gray bars represent the groups of sows with one, three and six parities, respectively.

After verification of seropositivity, a pool of peptides from representative nsp, structural proteins or a mix of both were used to evaluate antigen recognition by sera from sows with different numbers of parities. Figure 2 shows the ELISA absorbance values represented as the mean ± SD and values of each sow. Antibodies against peptides from nsp were low (absorbance ranges from 0 to 0.393), and no differences between groups of sows with different parities were observed. Antibodies to peptides of the structural proteins were high (absorbance ranges from 0 to 1.476); sows with six parities presented higher absorbance values compared with sows with one or three parities, although no significant differences were observed. A significant difference (*p* < 0.05) was observed when antibodies against nsp peptides (from all groups of sows) were compared with antibodies against structural peptides of sows with six parities. When the antibody response was evaluated against all the peptides (nsp and structural), no differences were observed between groups.

### 3.3. Frequency of Peptide-Specific IFN-γ Secreting Cells

**Figure 3:**
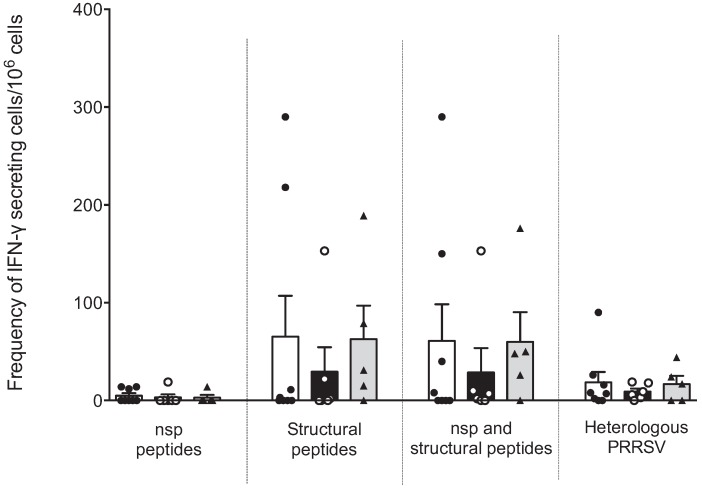
Cellular response against IFN-γ inducer peptides by multiparous and hyper-vaccinated sows. The IFNγ-SC frequencies between groups of sows against the pooled peptides from nsp and structural proteins of PRRSV were determined by ELISPOT IFN-γ assays of PBMCs. No statistical significance was observed in the IFNγ-SC frequency between sow groups. Each bar represents the mean ± standard deviation. White, black and gray bars represent the groups of sows with one, three and six parities, respectively.

Our next question was whether the cellular response, as evaluated by measuring the frequency of IFNγ-SC, differed between sows with one, three or six parities. Figure 3 shows that the number of IFNγ-SC against peptides from nsp was low or null; only a small number of sows responded. The response against peptides from structural proteins was higher than that of nsp, but a high variability was observed, and no significant differences were observed. No differences were observed between sows with different parities. In this experiment we used a heterologous virus to evaluate IFNγ-SC frequency (strain NVSL 97–7895). The response was low but less variability was observed.

**Figure 4:**
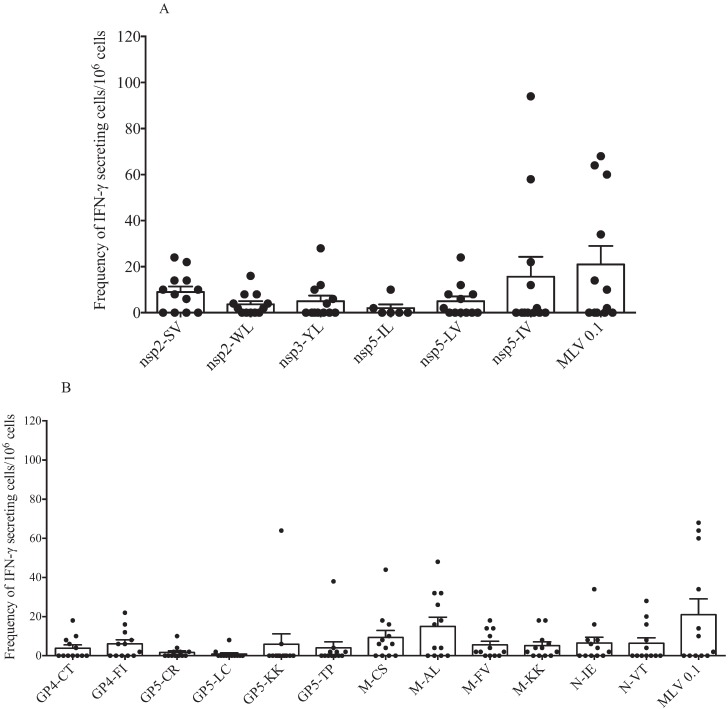
Cellular response against IFN-γ inducer peptides of both individual nsp and structural proteins by multiparous and hyper-vaccinated sows. IFNγ-SC frequencies of multiparous and hyper-immunized sows were evaluated by stimulating PMBCs of multiparous sows with IFN-γ inducer peptides from nsp (**A**) and structural proteins (**B**) of PRRSV. No statistical significance was observed in the IFNγ-SC frequency between sow groups. Each bar represents the mean of twelve sows ± standard deviation.

Because we did not observe differences in the number of IFNγ-SCs between sows with different parities, we evaluated the response to peptides of individual proteins (nsp and structural). Figure 4A,B shows the response of 12 sows of different parities (Table 1). As previously described, IFNγ-SCs were primarily induced in response to peptides from M protein; peptides from GP5 induced a lower response. Peptides from GP4 and N proteins induced a mid-level response. In this experiment, we used a homologous virus, and the response was variable; 50% of the sows showed a high response and 50% showed a null response. Figure 4B shows that response against nsp peptides was primarily directed to peptide nsp2-SV (60% of sows produced IFNγ-SC). The response to peptide nsp5-VI was variable and was observed in 40% of the sows. The response against the other peptides was highly variable, and no significant differences were observed.

## 4. Discussion

Vaccination has been frequently used to reduce PRRSV infection [7,8,9,13,19,20,21]. It is widely accepted that the MLV vaccine reduces viremia, mortality and virus shedding/replication, but cross-protection against heterologous viruses is partial [3,6,22]. Although one or two doses is enough to induce a sterilizing immunity in vaccinated animals [13,14], hyper-immunization or mass vaccination (three or more doses per animal per year) is common at some farms [11,12]. One interesting question is whether hyper-vaccination increases immunity. If it does, immunity against PRRSV and/or immunogenic peptides should be different between sows with differing numbers of parities. To answer this question, we evaluated the immune response of hyper-immunized sows against PRRSV and peptides that have been previously described as immunogenic [15,16,17,18].

Our results showed that the humoral response (evaluated through peptide recognition) to nps peptides was poor in all sows, independent of the number of parities. In contrast, the response observed against structural peptides was higher, especially in sows with six parities (*p* < 0.05). However, the increased response of sows with six parities compared with the response of sows with one and three parities was not significantly different. These data demonstrate that hyper-vaccination in sows increases the number of antibodies against structural peptides but not against nsp peptides. It is probable that peptides used in this study were poorly accessible to sows during vaccination or that the reduced viral loads due to each vaccination limited viral replication and thus contributed to the inaccessibility of nsp peptides to the immune system. Another explanation is that in this study we use nsp peptides from genotype I sequences (conserved and others non-conserved), which induce a low response in pigs immunized with PRRSV genotype II. In contrast, most of the structural peptides represent genotype II. Unfortunately, we were unable to evaluate the immune response against recombinant structural or nsp proteins, which would help in understanding the PRRSV immune response.

We evaluated the frequency of IFNγ-SC in sows with different parities in response to a mix of peptides from nsp, structural proteins or both. The results demonstrated a poor response to peptides from nsp in all sows and a variable response to the structural peptides and to the mix with all peptides. Additionally, we found that the frequency of peptide-specific IFNγ-SC was not different between sows with different numbers of parities, in contrast to the recognition of structural peptides by sera from sows with six parities *versus* sows with one parity. In spite of the low frequency of nsp peptide-specific IFNγ-SC observed in this study, this response was higher that the response previously reported to the same peptides; however, the previous study evaluated pigs immunized only once with type I or type II PRRSV vaccine [18]. This data suggest that hyper-vaccination could increase the frequency of nsp peptide-specific IFNγ-SC, but this response was lower than that observed against peptides from structural proteins.

Because we did not observe significant differences between sows with different numbers of parities, sows with different parities were also used to evaluate the response against individual peptides. As previously described, peptide nsp2-SV induced the most consistent response. In the case of nsp5-IV, some pigs showed a higher response, but in general the responses were quite variable, similar to the other peptides from nsp. These results confirm that nsp2 contains immunogenic peptides that can be recognized by memory cells induced in sows maintained in farms environments. The response observed against peptides from structural proteins was in agreement with previous reports using peptides [23] and others that describe M protein as the most immunogenic protein for inducing cellular responses [24]. Peptides M-AL and M-CS were the highest inducers of IFNγ-SC; the other peptides induced a variable response. It is important to note that variability observed in this in this study could be related to the haplotype of the sows, as has previously been reported by others [18,25].

The identification of T epitopes capable of inducing protective immunity is a constant challenge and represents a strategy for vaccine development. The evaluation of peptides that induce IFN-γ is a strategy used by others [17,25,26] and by us [18]. Previous reports evaluated these peptides under experimental conditions with infected or vaccinated pigs. In our study, we used samples from sows kept under farm conditions. This study design could be a disadvantage because the sows may have been exposed to other pathogens that affect the normal immune response. The sows used in this study appeared normal and had no sign of disease. In addition, reproductive parameters were normal and no significant differences were observed between sows with different number of parities. The use of sows kept under farm conditions is frequently used to evaluate vaccine programs against PRRSV and other viruses, and the results of these studies offer important information regarding farm immunity. The sows sampled in this study were in an Ingelvac^®^ PRRS-MLV vaccine program, suggesting that sows with more parities had also received more immunizations. This feature gave us the opportunity to analyze, in a natural system, how repeated immunizations increase the frequency of memory peptide-specific IFNγ-SC. Whether a hyper-vaccination program induces higher protective immunity in the herd is still an open question, and further studies are required. However, the recent description of a novel virulent strain in vaccinated herds [27] suggest that there are other important factors involved in the control of PRRSV, such as biosecurity [28].

## 5. Conclusions

In conclusion, this study demonstrates that sows with six parities that received 11–13 vaccinations showed increased antibodies against peptides from structural proteins but not against peptides from nsp. This conclusion is supported by the significant difference observed between sows with six parities *versus* sows with one parity. In contrast, we did not observe differences between parities when the frequency of IFNγ-SC was evaluated; we only confirmed that nsp2-SV and peptides from M proteins were the best inducers of IFNγ-SC. Further studies are needed to confirm whether these peptides or others can be targets for vaccine development.
